# Structural substitutions on the methoxybenzene ring retain the biological activity of the zaxinone mimics MiZax3

**DOI:** 10.3389/fpls.2025.1631066

**Published:** 2025-07-18

**Authors:** Jian You Wang, Muhammad Jamil, Lamis M. Tareq Berqdar, Ikuo Takahashi, Tsuyoshi Ota, Tadao Asami, Salim Al-Babili

**Affiliations:** ^1^ The BioActives Lab, Center for Desert Agriculture, King Abdullah University of Science and Technology, Thuwal, Saudi Arabia; ^2^ Kihara Institute for Biological Research, Yokohama City University, Yokohama, Kanagawa, Japan; ^3^ Plant Science Program, Biological and Environmental Science and Engineering Division, King Abdullah University of Science and Technology (KAUST), Thuwal, Saudi Arabia; ^4^ Center of Excellence – Sustainable Food Security, King Abdullah University of Science and Technology (KAUST), Thuwal, Saudi Arabia

**Keywords:** zaxinone, zaxinone mimics, MiZax, apocarotenoids, strigolactones, rice (*Oryza sativa*), biostimulant

## Abstract

The plant growth regulator zaxinone is essential for proper rice growth and development. Additionally, zaxinone and its two synthetic mimics, MiZax3 and MiZax5, have been shown to significantly promote crop growth and reduce infestation by the root parasitic plant *Striga* by suppressing strigolactone (SL) production, highlighting their potential for field application. Here, we developed 4 new MiZax through structural modifications of the methoxybenzene ring in MiZax3 and evaluated their effects on plant growth and SL exudation. These newly developed mimics enhanced rice growth and reduced SL release without compromising the bioactivity of the lead compound MiZax3. Our findings underscore their potential to guide future chemical design efforts aimed at exploring zaxinone biology.

## Introduction

The expanding global population exerts unprecedented pressure on food demand, which is expected to intensify the risks of starvation, malnutrition, and food insecurity in the future (Hu et al., 2023). In response to this growing challenge, the United Nations Food and Agriculture Organization (FAO) estimates that global food production must nearly triple by 2050 to meet the nutritional needs of an expanding human population (FAO: The World Needs 70% More Food by 2050)^a^. One of the most viable strategies to address this urgent issue is to enhance crops yield. Central to this strategy is a deeper understanding of plant physiological responses, particularly how plants use metabolites as chemical signals to regulate growth and adapt to changing environmental conditions, such as nutrient availability in the soil (Weng et al., 2021; Hu et al., 2023; Xu et al., 2023). These metabolites play a crucial role in mediating plant interactions within the rhizosphere. In response to environmental stimuli, plants exude specific metabolites that facilitate communication with neighboring plants, microbes, and parasitic organisms. This exudation process supports plant adaptation and survival under both abiotic and biotic stress conditions (Massalha et al., 2017; Pang et al., 2021; Weng et al., 2021). A significant proportion of these signaling molecules are derived from secondary metabolic pathways, such as carotenoid biosynthesis. Indeed, the cleavage of carotenoids is particularly interesting, which gives rise to precursors of evolutionarily conserved plant hormones such as abscisic acid and strigolactones (SLs) as well as the apocarotenoid signaling molecules, zaxinone and anchorene (Al-Babili and Bouwmeester, 2015; Wang et al., 2019; Jia et al., 2019; Wang et al., 2021b).

**Figure 1:**
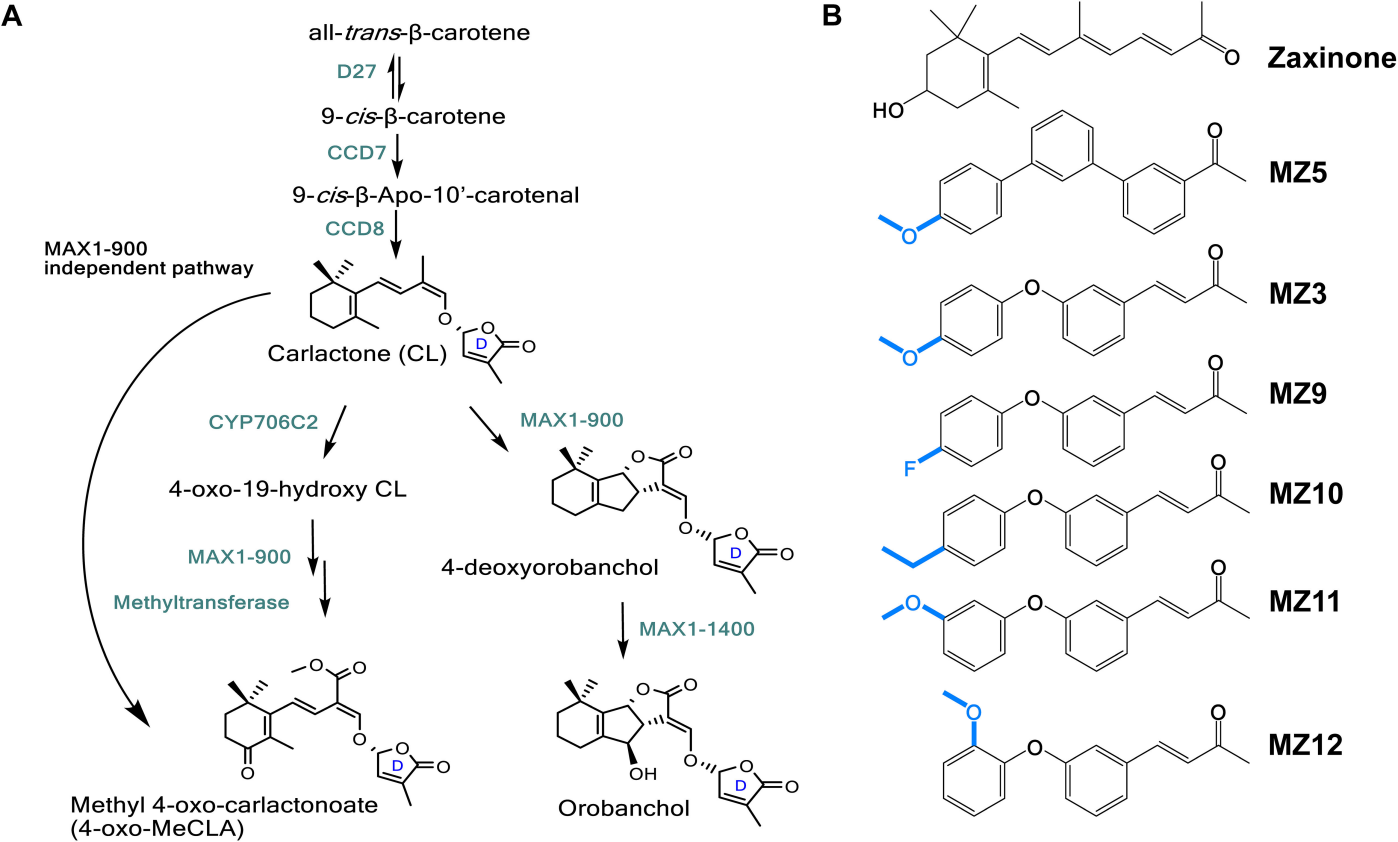
Scheme of strigolactone (SL) biosynthesis in rice and the stricture of zaxinone mimics (MiZax). **(A)** SL biosynthesis involves the sequential action of several key enzymes: the isomerase DWARF27 (D27), two carotenoid cleavage dioxygenases (CCD7 and CCD8), and cytochrome P450 enzymes of the CYP711 clade. D27 catalyzes the reversible isomerization of all-*trans*-β-carotene into 9-*cis*-β-carotene, which is then converted by CCD7 and CCD8 into carlactone (CL), a central intermediate in SL biosynthesis. Canonical and non-canonical SLs are subsequently formed through further enzymatic modifications of CL by various cytochrome P450s, such as CYP711A (MORE AXILLARY GROWTH1) and CYP706C. **(B)** Chemical structures of MiZax (MZ) compounds, including zaxinone, MZ5, the parent compound MZ3 and its derivatives MZ9, MZ10, MZ11, and MZ12. Blue colors indicate the chemical substitutions.

SLs have garnered significant attention due to their multifaceted roles in plant development, stress adaptation, and rhizosphere interactions (Al-Babili and Bouwmeester, 2015; Lanfranco et al., 2018; Fiorilli et al., 2019; Wang et al., 2024a). The biosynthetic pathway of SLs begins with the reversible isomerization of all-*trans*-β-carotene into 9-*cis*-β-carotene, a reaction catalyzed by the isomerase enzyme DWARF27 (Alder et al., 2012; Abuauf et al., 2018). This is followed by sequential oxidative cleavage and molecular rearrangement reactions carried out by carotenoid cleavage dioxygenases CCD7 and CCD8, resulting in the production of carlactone (CL), a key intermediate in SL biosynthesis (Alder et al., 2012; Abe et al., 2014; Bruno and Al-Babili, 2016; Bruno et al., 2017; Chen et al., 2022). CL serves as a substrate for cytochrome P450 (CYP450) monooxygenases, including enzymes from the CYP711A and CYP706C2 subfamilies, which further metabolize CL into canonical or non-canonical SLs (Ito et al., 2022; Chen et al., 2023; Li et al., 2024; Wang et al., 2024a, b; Zhang et al., 2014) (**Figure 1A**).

We recently identified the apocarotenoid regulatory metabolite zaxinone (structure shown in **Figure 1B**), which promotes plant growth, enhances sugar metabolism, and suppresses SL biosynthesis in rice, thereby reducing *Striga* infestation under greenhouse conditions (Wang et al., 2019, 2021a, 2023a). However, the complex and costly synthesis of zaxinone limits practical application. To overcome this, we developed two synthetic mimics, MiZax3 and MiZax5 (**Figure 1B**), which highly replicate the effects of zaxinone in terms of rice growth and SL suppression (Wang et al., 2020). In addition to enhancing growth and yield in several horticultural crops under open-field conditions (Wang et al., 2022a, 2023b; Jalal et al., 2025), these mimics showed no adverse effects on soil microbial communities (Mazzarella et al., 2024), highlighting their potential as environmentally friendly biostimulants. Furthermore, we developed MiZax6, MiZax7, and MiZax8 by introducing modifications to the carbonyl-containing moiety, the ketone group, of MiZax3 to increase hydrophobicity and potentially enhance uptake (Jamil et al., 2023). Intriguingly, altering the ketone group did not affect the compounds’ activity on root growth or SL biosynthesis and release, suggesting that replacement of this functional group maintained the biological activity (Jamil et al., 2023); however, whether modifications of the methoxybenzene ring would change the biological activities remain elusive (**Figure 1B**). To test this, we designed and developed a series of MiZax compounds, MiZax9-12, with substitutions on the methoxybenzene ring and evaluated their biological activities in plants.

## Materials and methods

### Plant material and growth conditions

WT Nipponbare rice plants were grown under controlled conditions (a 12 h photoperiod, 200-µmol photons m^−2^ s^−1^ and day/night temperature of 27/25°C). Rice seeds were surface-sterilized in a 50% sodium hypochlorite solution with 0.01% Tween-20 for 15 min. Seeds were then rinsed with sterile water and germinated in the dark overnight. The pre-germinated seeds were transferred to Petri dishes containing half-strength liquid Murashige and Skoog (MS) medium and incubated in a growth chamber for 7 days. Thereafter, the seedlings were transferred into black falcon tubes filled with low-Pi of half-strength modified Hoagland nutrient solution with adjusted pH to 5.8. The nutrient solution consisted of 5.6 mM NH_4_NO_3_, 0.8 mM MgSO_4_·7H_2_O, 0.8 mM K_2_SO_4_, 0.18 mM FeSO_4_·7H_2_O, 0.18 mM Na_2_EDTA·2H_2_O, 1.6 mM CaCl_2_·2H_2_O, 0.8 mM KNO_3_, 0.023 mM H_3_BO_3_, 0.0045 mM MnCl_2_·4H_2_O, 0.0003 mM CuSO_4_·5H_2_O, 0.0015 mM ZnCl_2_, 0.0001 mM Na_2_MoO_4_·2H_2_O and 0.004 mM K_2_HPO_4_·2H_2_O.

### Synthesis of MZ9 to MZ12

Detailed synthetic method is available in **Supplementary Document 1**.

### Plant phenotyping

For phenotyping, 1 week-old rice seedlings were transferred into 50 mL tubes, filled with half-strength modified Hoagland nutrient solution with 5.8 pH. Seedlings were treated with 1.0 µM MiZax (solved in 0.1% acetone) or the corresponding volume of the solvent (control) for two weeks. The solution was refreshed at two days intervals.

### SL quantification in root exudate

The SL analysis in rice root exudates was performed according to the protocol published in (Wang et al., 2022c). For this purpose, rice plants were grown hydroponically in 50 mL tubes for two weeks under low phosphate conditions and treated with 5.0 μM MiZax (solved in 0.1% acetone) or the corresponding volume of the solvent (control) for 6 h. SLs were then collected from root exudates. Briefly, 50 mL of root exudates spiked with 0.672 ng of 20 ng *rac*-GR24 was brought on a C_18_-Fast Reversed-Phase SPE column (500 mg 3 mL^-1^) preconditioned with 3 mL of methanol and 3 mL of water. After washing with 3 mL of water, SLs were eluted with 5 mL of acetone. The SLs fraction was concentrated to SL aqueous solution (∼1 mL), followed by 1 mL of ethyl acetate extraction. 750 μL of SL enriched organic phase was dried under vacuum. The final extract was re-dissolved in 100 μL of acetonitrile: water (25:75, v:v) and filtered through a 0.22 μm filter for LC-MS/MS analysis.

SLs were quantified by LC-MS/MS using a UHPLC- Triple-Stage Quadrupole Mass Spectrometer (Thermo Scientific™ Altis™). Chromatographic separation was the same as above SL identification. The MS parameters were: positive ion mode, ion source of H-ESI, ion spray voltage of 5000 V, sheath gas of 40 arbitrary units, aux gas of 15 arbitrary units, sweep gas of 2 arbitrary units, ion transfer tube gas temperature of 350°C, vaporizer temperature of 350 °C, collision energy of 17 eV, CID gas of 2 mTorr. The characteristic Multiple Reaction Monitoring (MRM) transitions (precursor ion → product ion) were 331.15→216.0, 331.15→234.1, 331.15→97.02 for 4-deoxyorobanchol; 347.14→329.14, 347.14→233.12, 347.14→ 205.12, 347.14→97.02 for orobanchol; 361.16→ 247.12, 361.16→177.05, 361.16→208.07, 361.16→97.02 for 4-oxo-MeCLA; 299.09→185.06, 299.09→157.06, 299.09→97.02 for GR24. 317.17→ 220.14, 317.17→205.12, 317.17→164.08, 317.17→97.02 for CL+14 (putative oxo-CL).

### Striga germination bioassays

Assays were performed as the published procedure (Jamil et al., 2023). Rice plants were grown hydroponically in 50 mL tubes for two weeks under low-phosphate conditions and treated with 5.0 µM MiZax for 6 h. SLs were extracted from root exudates using C18 columns and applied to pre-conditioned Striga seeds. For pre-conditioning, Striga seeds were surface-sterilized with 50% bleach for 5 min, rinsed six times with sterile Milli-Q water, and air-dried. Approximately 50–100 seeds were placed on 9 mm glass fiber filter paper discs. Twelve discs were transferred to Petri dishes containing moistened Whatman paper, sealed, and incubated at 30°C in darkness for 10 days. The pre-conditioned Striga seeds were treated with 55 µL of each sample and incubated at 30°C for 24 h. Germinated and total seeds were counted using SeedQuant (Braguy et al., 2021) to calculate germination percentages.

### Statistical analysis

Data are represented as mean and their variations as standard deviation. The statistical significance was determined by one-way analysis of variance (one-way ANOVA) with Tukey’s multiple comparison test, using a probability level of p<0.05; or two-tail student t-test with denote significant differences (*p < 0.05, **p< 0.01, ***p< 0.001, ****p< 0.0001). All statistical elaborations were performed using GraphPad Prism, version 8.3.0.

## Results and discussion

### Modification of MiZax3 methoxybenzene ring shows bioactivity in rice

**Figure 2:**
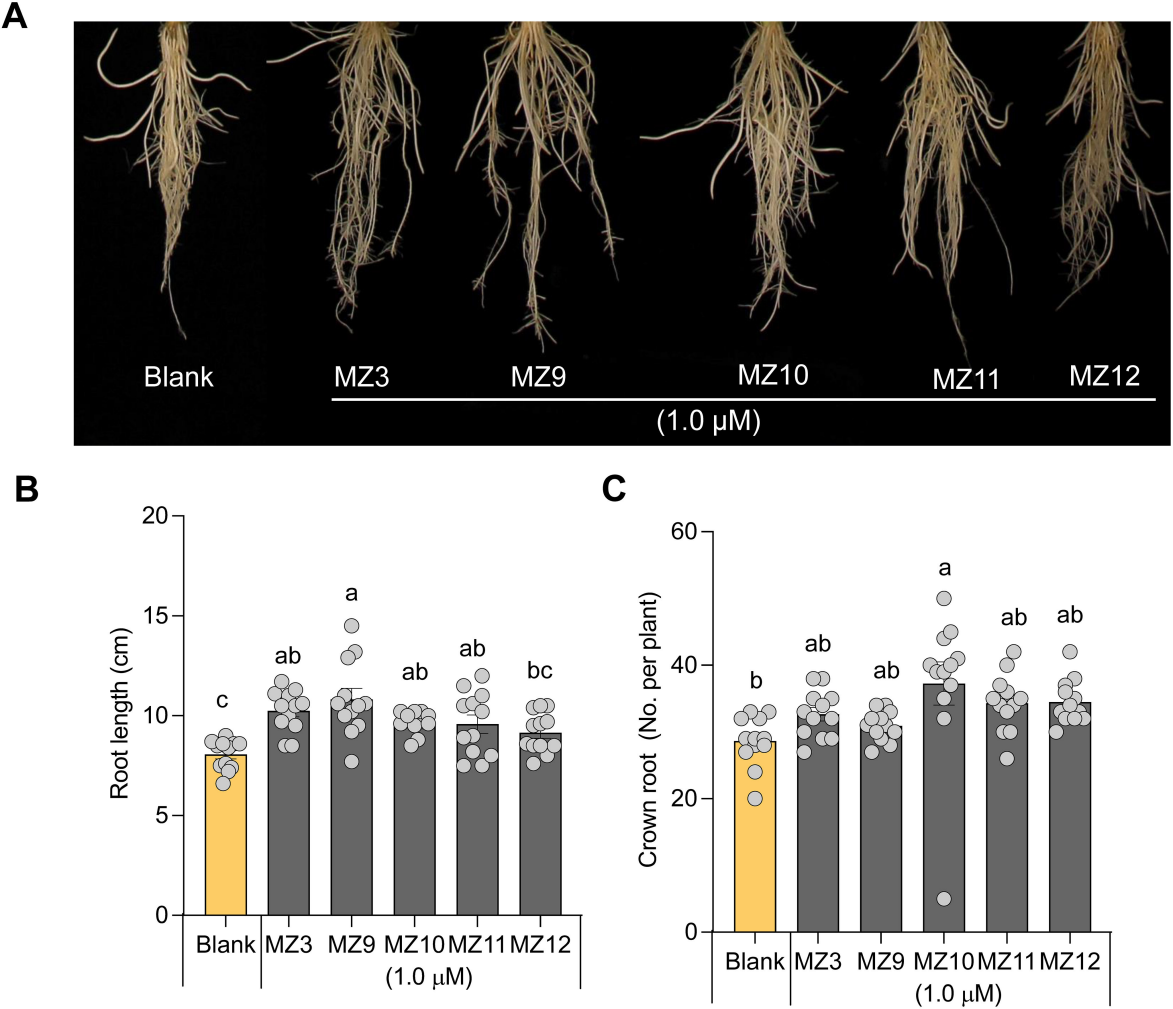
Effect of MZ9, MZ10, MZ11, and MZ12 on rice root growth and development. **(A)** Representative image of rice seedlings treated with different MiZax. **(B)** Quantitative effect of MZ9–MZ12 on root length. **(C)** Effect of MZ9–MZ12 on crown root number. Compounds were applied at a concentration of 1.0 µM to hydroponically grown rice seedlings for two weeks. Data represent means ± SE (n = 12). Statistical analysis was performed using one-way ANOVA followed by Tukey’s *post hoc* test. Different letters indicate statistically significant differences (P < 0.05). MZ, MiZax.

**Figure 3:**
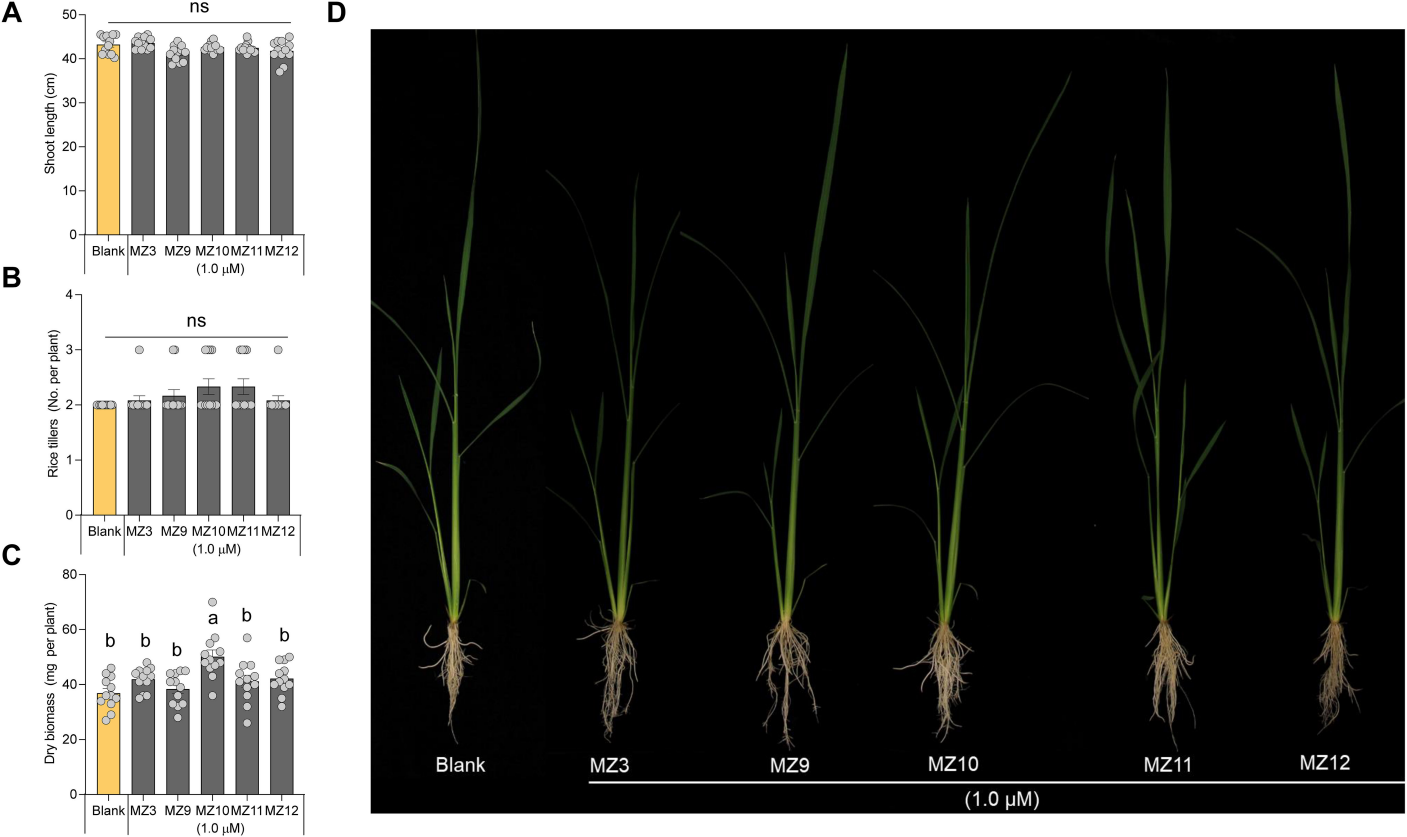
Effect of MZ9, MZ10, MZ11, and MZ12 on rice shoot growth and development. Quantitative effect of MZ9–MZ12 on **(A)** shoot length, **(B)** number of tillers, and **(C)** shoot dry biomass. **(D)** Representative image of rice seedlings treated with different MiZax analogs. Compounds were applied at a concentration of 1.0 µM to hydroponically grown rice seedlings for two weeks. Data represent means ± SE (n = 12). Statistical analysis was conducted using one-way ANOVA followed by Tukey’s *post hoc* test. Different letters indicate statistically significant differences (P < 0.05). MZ, MiZax.

To investigate the impact of structural modifications to the methoxybenzene ring on biological activity, we designed and synthesized compounds MZ9 through MZ12. In these analogs, the para-methoxy group of MZ3 was substituted with a fluoro, ethyl, meta-methoxy, or ortho-methoxy group, respectively (**Figure 1B**). To assess the activity of the new mimics, we used MZ3 as reference, which showed promising effects at low concentrations under both hydroponic (1.0 µM) and open-field conditions (2.5 µM) (Wang et al., 2020; Wang et al., 2022a; Wang et al., 2023b; Jamil et al., 2023), we tested the new mimics at these concentrations. We, evaluated their growth-promoting effects at a concentration of 1.0 µM on hydroponically grown rice seedlings, using MZ3 as a positive control. All four compounds exhibited significant growth-enhancing activity, leading to a 30% to 50% increase in root length and a 10% to 20% increase in crown root number (**Figures 2B, C**). Notably, only MZ10 significantly improved shoot biomass (**Figure 3C**), showing the most pronounced overall effect with an approximately 20% increase in crown root number and a 40% enhancement in shoot biomass.

Moreover, it is worth noting that the growth-promoting effects of these compounds were more pronounced in roots than in shoots, consistent with previous findings on earlier generations of MiZax (Wang et al., 2020; Jamil et al., 2023). The positive control, MZ3, displayed comparable activity to MZ9–MZ12 in promoting root length, crown root number, shoot length, and tiller development (**Figures 2**, **3**).

We recently reported the growth-promoting effects of zaxinone and its synthetic mimics, MiZax, with a particular emphasis on root growth and development in rice (Wang et al., 2020; Jamil et al., 2023). The promising outcomes from these studies highlight the potential utility of these biostimulants for field applications aimed at improving several crop performance and productivity (Wang et al., 2020, 2022a; Wang et al., 2023b; Jalal et al., 2025). Previous studies showed that substituting the ketone group of MZ3 with imine, alcohol, or ether moieties enhanced root biomass without causing adverse effects, suggesting that while the ketone contributes to bioactivity, its position allows for a degree of structural flexibility (Jamil et al., 2023). In the present study, we developed a new series of MiZax by introducing structural modifications to the methoxybenzene ring of MiZax3, aiming to investigate their impact on biological activity.

The substitution of the para-methoxy group in MZ3 with fluoro, ethyl, meta-methoxy, or ortho-methoxy groups resulted in similar positive effects on root growth and an increase in the number of crown roots in rice seedlings, comparable to MZ3. This suggests that the “-O-” group and the position of the methoxy group do not significantly restrict bioactivity. Notably, the ethyl substitution (MZ10) exhibited slightly enhanced activity compared to MZ3, particularly in regulating crown root development and shoot biomass. This suggests that elongation on the alkyl chain could enhance the growth-promoting activity.

### MZ9, MZ10, MZ11, and MZ12 are negative regulators of SL release in rice

**Figure 4:**
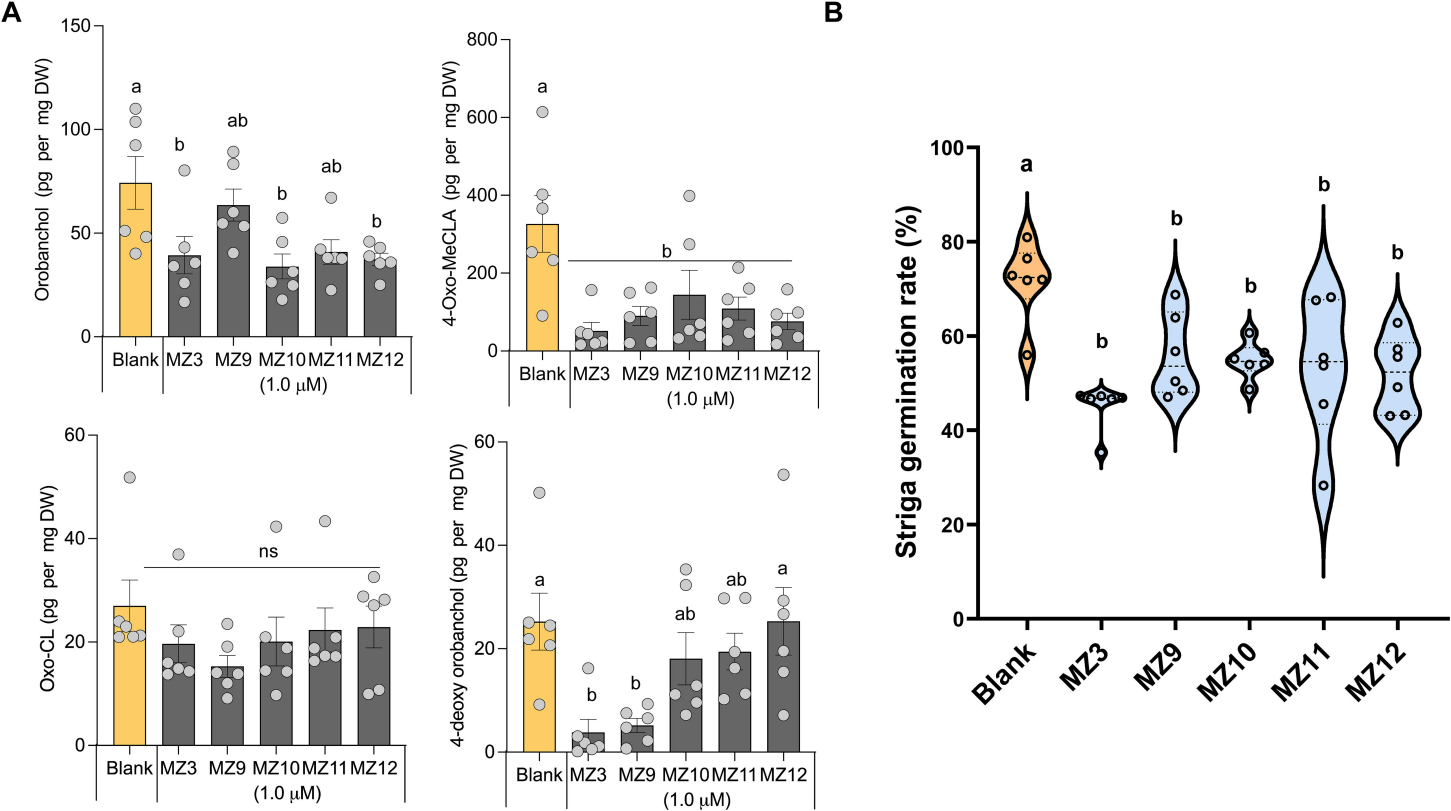
MZ9, MZ10, MZ11, and MZ12 on rice strigolactone release. **(A)** SL levels in rice root exudates following treatment with MZ9–MZ12 were quantified using LC-MS. **(B)** Striga seed germination assays were conducted using root exudates from the respective treatments. All compounds were applied at a concentration of 5.0 µM to two-week-old hydroponically grown rice seedlings under phosphorus-deficient conditions. Data represent means ± SE (n = 6). Statistical analysis was performed using one-way ANOVA followed by Tukey’s *post hoc* test. Different letters indicate statistically significant differences (P < 0.05). MZ, MiZax; ns, not significant.

Next, we assessed the effects of MZ9–MZ12 on SL content in rice root exudates, which is usually measured after a 6-hour incubation period (Wang et al., 2019; Ito et al., 2022; Jamil et al., 2023). Treatment with these compounds at a 5.0 µM concentration led to a significant reduction in SL levels, as confirmed by LC–MS analysis Specifically, the canonical SL orobanchol levels decreased by 20% to 50%, while methyl 4-oxo-carlactonoate (4-oxo-MeCLA), a non-canonical SL, was reduced by 34% to 78%. In contrast, 4-deoxyorobanchol, the second major canonical SL in rice, was significantly suppressed only by MZ9. Notably, none of the compounds markedly affected the levels of tentative oxo-carlactone (oxo-CL) (Wang et al., 2022b) (**Figure 4A**). The effects were consistently validated through a Striga bioassay, which demonstrated a reduction of approximately 30% in Striga seed germination compared to the blank treatment (**Figure 4B**). Similarly, the positive control, MZ3, resulted in a reduction of approximately 45% in orobanchol levels, 80% in 4-deoxyorobanchol, 85% in 4-oxo-MeCLA, and 40% in Striga seed germination (**Figures 4A, B**).

The apocarotenoid hormone SLs not only regulates developmental processes that shape shoot and root architecture but also facilitate the colonization of host plants by symbiotic fungi (Al-Babili and Bouwmeester, 2015; Lanfranco et al., 2018). However, the quantity and composition of SLs released by host roots into the rhizosphere are directly related to the infection rate of root parasitic weeds (Wang et al., 2024a). Therefore, reducing the secretion of SLs could potentially lower the infection rates of root parasitic plants in the rhizosphere (Kuijer et al., 2024). In our study, treatment with MZ9–MZ12 generally led to a reduction in the levels of various SLs, particularly orobanchol and 4-oxo-MeCLA, while having minimal impact on the amount of oxo-CL. However, most of the reduced SLs in this study are weak germination stimulants for Striga. Indeed, several studies have demonstrated that 4-deoxyorobanchol is a stronger germination stimulant for Striga seeds than orobanchol and 4-oxo-MeCLA in rice (Ito et al., 2022; Chen et al., 2023; Li et al., 2024). Notably, the suppressive effects of MiZax on SL levels varied depending on the specific SL type affected, suggesting that certain MiZax may be particularly valuable not only for mitigating *Striga* infection but also for modulating beneficial arbuscular mycorrhizal symbiosis (Lanfranco et al., 2018; Wang et al., 2024a). In this study, we observed that the fluoro substitution in the methoxybenzene ring (MZ9) significantly decreased the level of 4-deoxyorobanchol without impacting the downstream metabolite orobanchol. Conversely, substitutions with ethyl, meta-methoxy, or ortho-methoxy groups (MZ10–12) did not affect 4-deoxyorobanchol levels but led to a suppression of orobanchol accumulation. These findings suggest that the functional groups present on the methoxybenzene ring may selectively affect the expression or the enzymatic activity of MAX1–900 or MAX1-1400 (**Figure 1A**). Further investigations are warranted to validate this hypothesis.

Taken together, our results further demonstrate that structural modifications to the methoxybenzene ring of the previously developed MiZax3 did not diminish bioactivities, particularly in promoting rice growth. This study thus provides valuable insights to inform future chemical design and highlights potential targets for research on SL-related pathways as well as zaxinone biology.

## Data Availability

The original contributions presented in the study are included in the article/**Supplementary Material**. Further inquiries can be directed to the corresponding author.

## Footnotes

a
https://www.isaaa.org/kc/cropbiotechupdate/article/default.asp?ID=4778pa
