# Supplementary material for: Structural substitutions on the methoxybenzene ring retain the biological activity of the zaxinone mimics MiZax3
Source: Front Plant Sci. 2025 Jul 18;16:1631066. doi: 10.3389/fpls.2025.1631066 (PMC12313581; doi:10.3389/fpls.2025.1631066)
Supplement: Supplementary file 1 [file Presentation1.pdf]

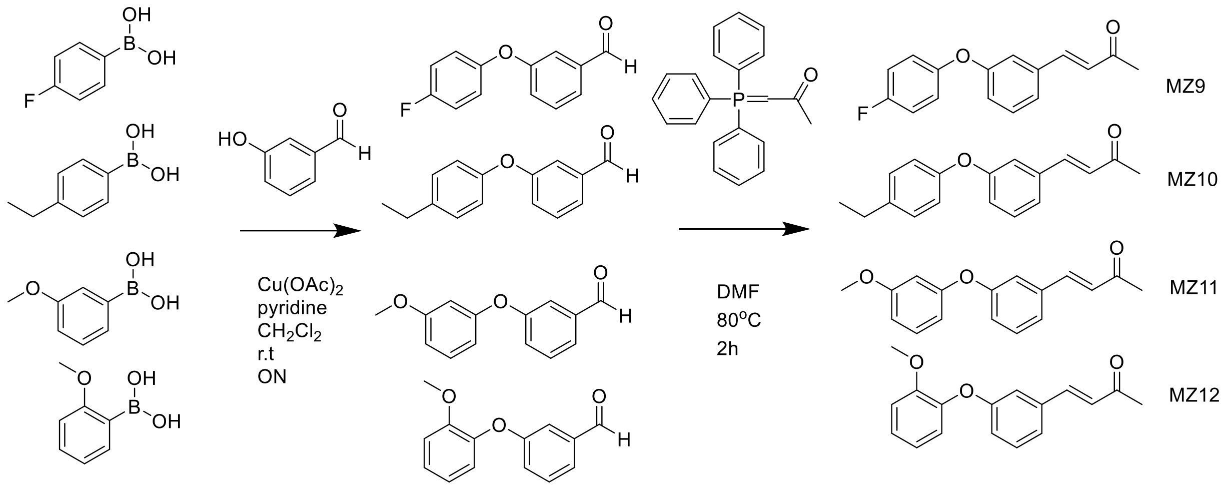

General synthetic procedure

A round-bottom flask was charged with boronic acid (3 mmol), 3-hydroxybenzaldehyde (1 mmol), pyridine (8 mmol), and $Cu(OAc)_2$ (4 mmol) in dichloromethane (10 mL) as the solvent. The reaction mixture was stirred at room temperature overnight. After completion of the reaction, the mixture was diluted with ethyl acetate and successively washed with 1 N NaOH, 1 N HCl, and brine. The organic layer was dried over sodium sulfate, filtered, and concentrated under reduced pressure. The resulting residue was purified by silica gel column chromatography (Wakosil® C-300HG, hexane:ethyl acetate) to afford the desired compound.

The entire amount of the obtained product was used in the next reaction without further purification.

To a round-bottom flask were added the aldehyde (1 mmol), (acetylmethylene)triphenylphosphorane (4 mmol), and DMF (2 mL) as the solvent. The mixture was stirred at 80 ^{o}C for 2 hours. After completion of the reaction, the crude mixture was directly purified by silica gel column chromatography (Wakosil® C-300HG, hexane:ethyl acetate) to afford the desired compound.

Besides, IR spectra were measured on Jasco FT/IR-4200 spectrometer (ATR) while the melting points were measured on Yanaco micro melting point apparatus.

- MZ9

2 steps yield 33%

1H-NMR (500 MHz, CDCl3) δ: 7.44 (d, J = 16 Hz, 1H), 7.35 (t, J = 8.0 Hz, 1H) , 7.26 (d, J = 8.0 Hz, 1H) , 7.11 (s, 1H), 7.03-7.09 (m, 2H) , 6.97-7.02 (m, 3H), 6.65 (d, J = 16.0 Hz, 1H) , 2.37 (s, 3H). 13C-NMR (126 MHz, DMSO-d6) δ:198.19, 159.05 (d, J = 242.8 Hz), 158.32, 152.17 (d, J = 2.5 Hz), 142.54, 136.23, 130.28 127.73, 123.02, 120.87 (d, J = 8.8 Hz), 120.03, 117.03, 116.49(23.9 Hz). HRMS-Esi: m/z [M-Na]$^{-}$ Calcd for $C_{15}H_{14}Na_{1}O_{5}$:297.07389, found: 297.07252. IR(ATR): 1667, 1498 cm^{-1}. Melting point: 50-51 ^{o}C.

MZ9
1H: MZ9_TO816C1_1HNMR

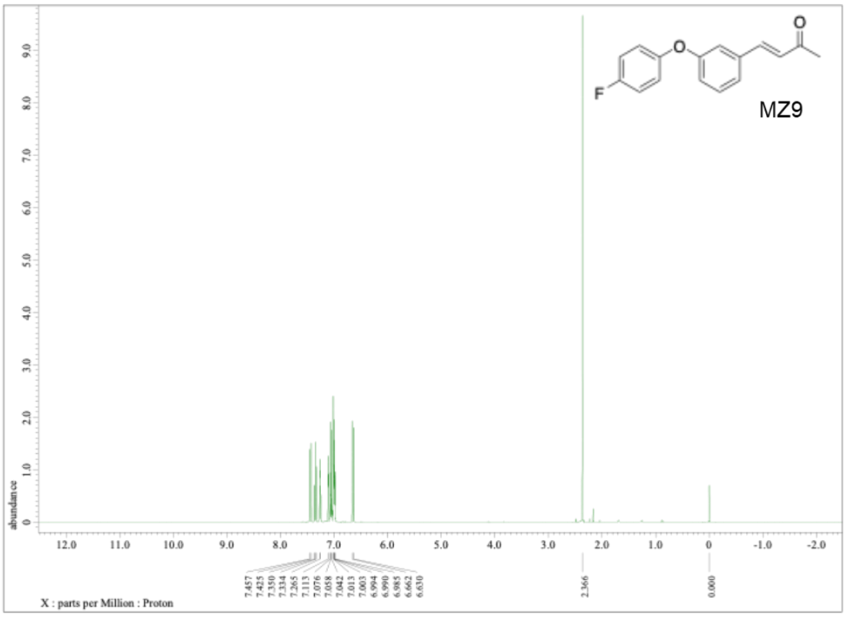

MZ9
13C: MZ9_TO816C1_13CNMR

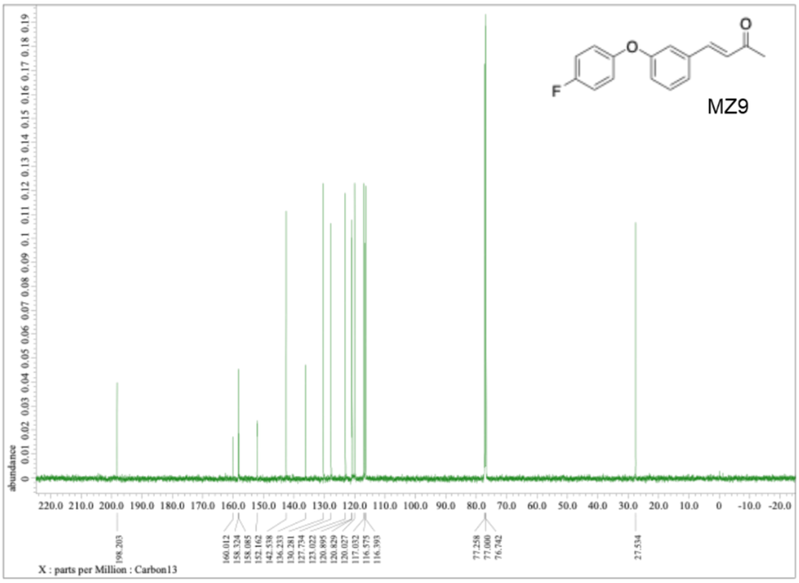

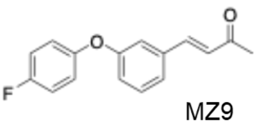

MZ9

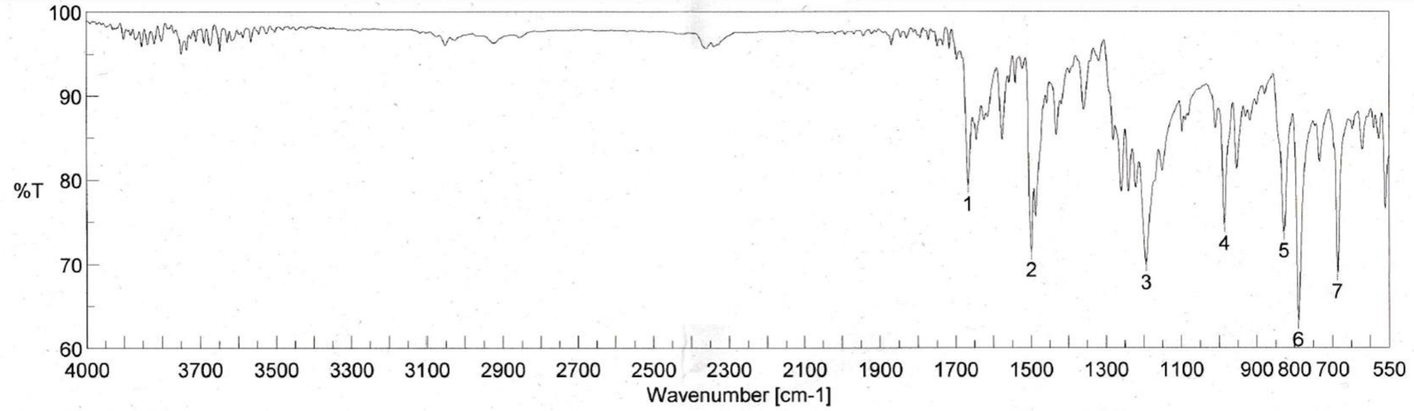

IR spectrum of MZ9.

- MZ10

2 steps yield 36%

1H-NMR (500 MHz, CDCl3) δ: 7.44 (d, J = 16.5 Hz, 1H), 7.33 (t, J = 7.5 Hz, 1H), 7.23 (d, J = 7.5 Hz, 1H) , 7.18 (d, J = 8.5 Hz, 2H) , 7.13 (s, 1H), 7.02 (d, J = 7.5 Hz, 1H) , 6.95 (d, J = 8.5 Hz, 2H) , 6.64 (d, J = 16.5 Hz, 1H) , 2.65 (q, J = 7.5 Hz, 2H) , 2.35 (s, 1H) , 1.25 (t, J = 7.5 Hz, 3H).
13C-NMR (126 MHz, DMSO-d6) δ: 198.25, 158.41, 154.12, 142.78, 139.84, 136.05130.12, 129.16(2C), 127.58, 122.1, 120.24, 119.30(2C), 117.13, 28.11, 27.44, 15.62.
HRMS-Esi: m/z [M-Na]- Calcd for $C_{15}H_{14}Na_{1}O_{5}$:297.07389, found: 297.07252. IR(ATR): 2964, 1669, 1505 cm^{-1}. Oil.

MZ10
1H: MZ10_TO816D1_1HNMR

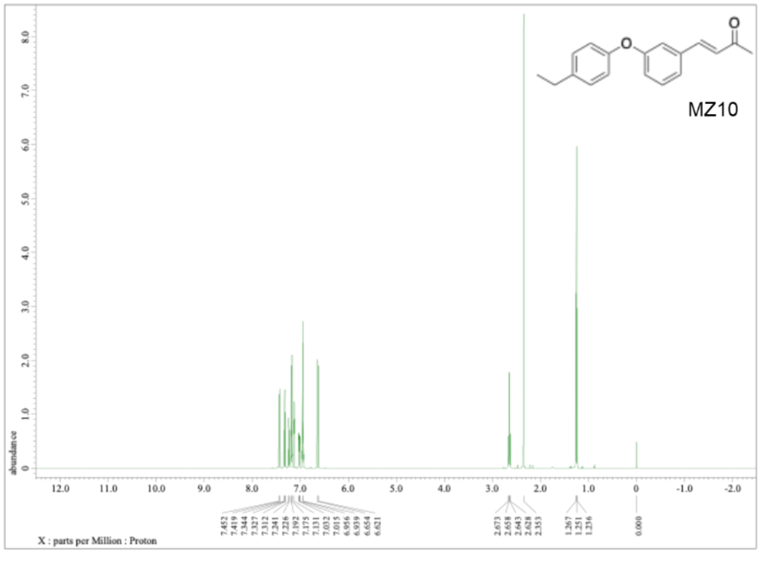

MZ10
13C: MZ10_TO816D1_13CNMR

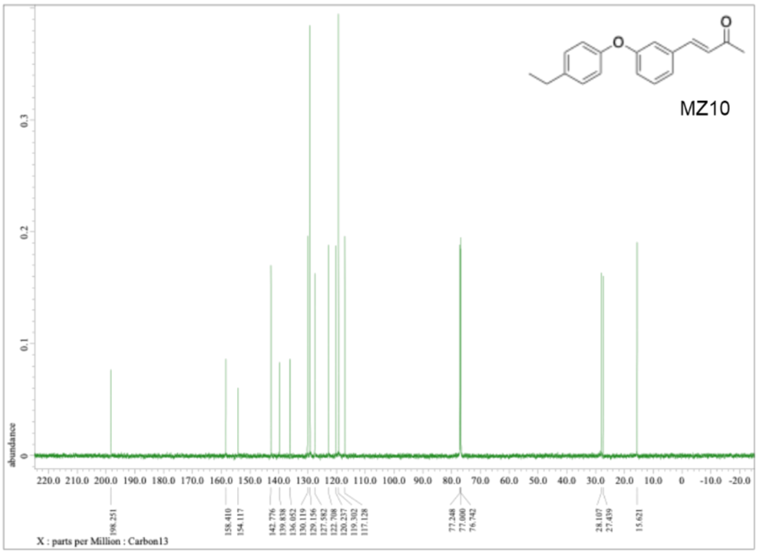

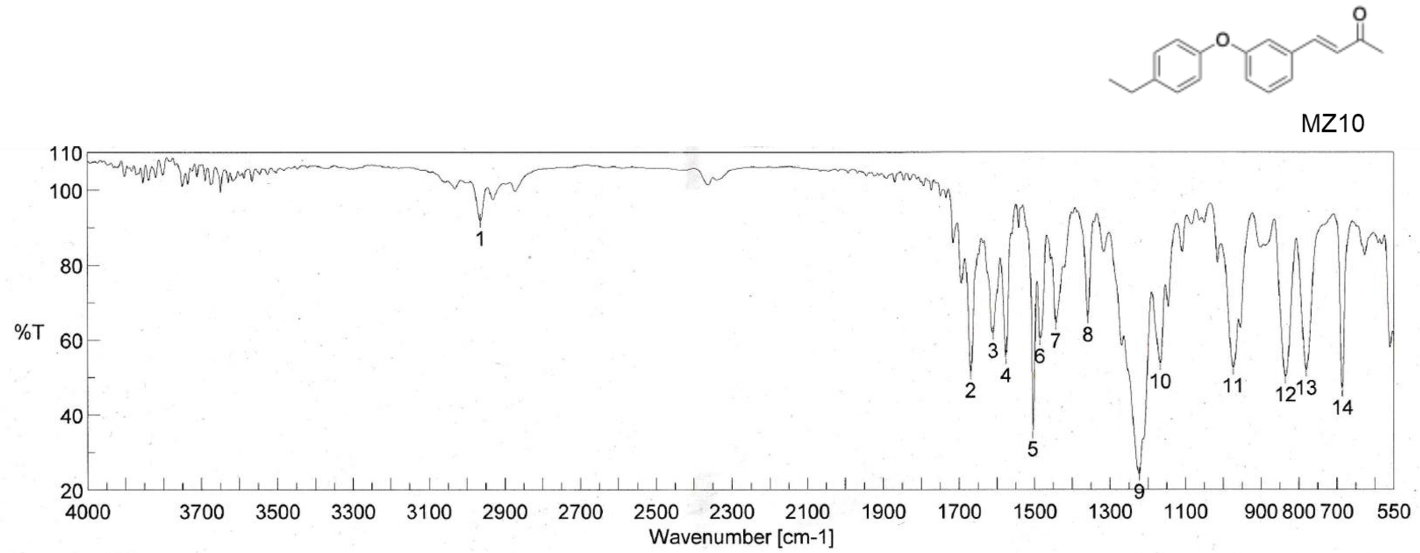

IR spectrum of MZ10.

- MZ11

2 steps yield 33%

1H-NMR (500 MHz, CDCl3) δ: 7.45 (d, J = 16.0 Hz, 1H), 7.36 (t, J = 8.0 Hz, 1H), 7.22-7.29 (m, 2H) , 7.17 (s, 1H), 7.06 (d, J = 8.5 Hz, 1H), 6.69 (d, J = 8.5 Hz, 1H) , 6.65 (d, J = 16.0 Hz, 1H) , 6.57-6.62 (m, 2H) , 3.79 (s, 3H), 2.36 (s, 3H).
13C-NMR (126 MHz, DMSO-d6) δ: 198.24, 161.00, 157.77, 157.70, 142.64, 136.20, 130.29, 130.24, 127.70, 123.24, 120.84, 117.85, 111.20, 109.28, 105.24, 55.36, 27.54.
HRMS-Esi: m/z $[M-Na]^-$ Calcd for $C_{15}H_{14}Na_1O_5$:297.07389, found: 297.07252. IR(ATR): 1667, 1486 cm^{-1}. Melting point: 35.5-36.5 ^{o}C.

MZ11
1H: MZ11_TO816A1_1HNMR

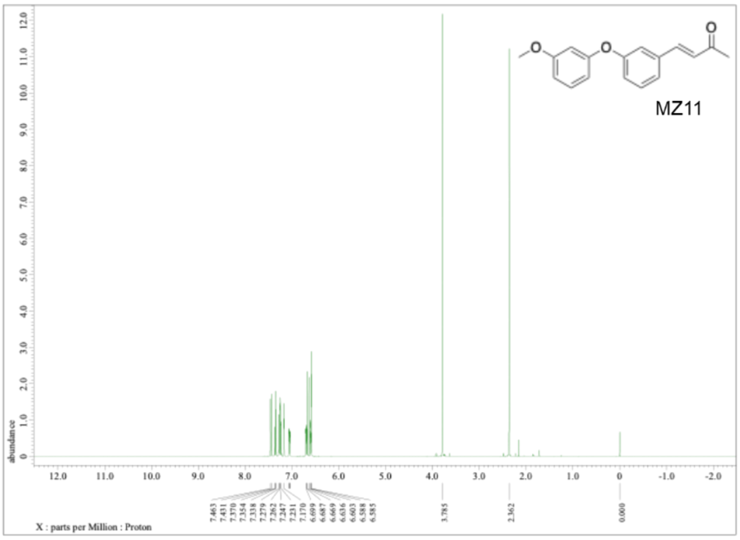

MZ11
13C: MZ11_TO816A1_13CNMR

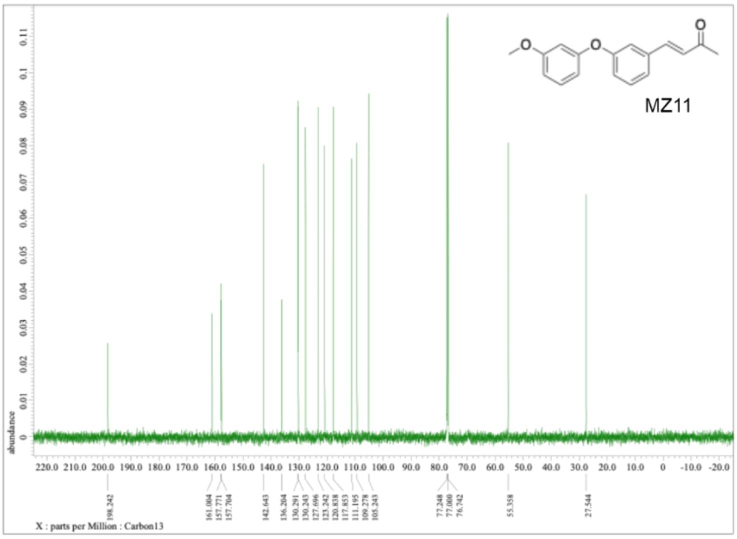

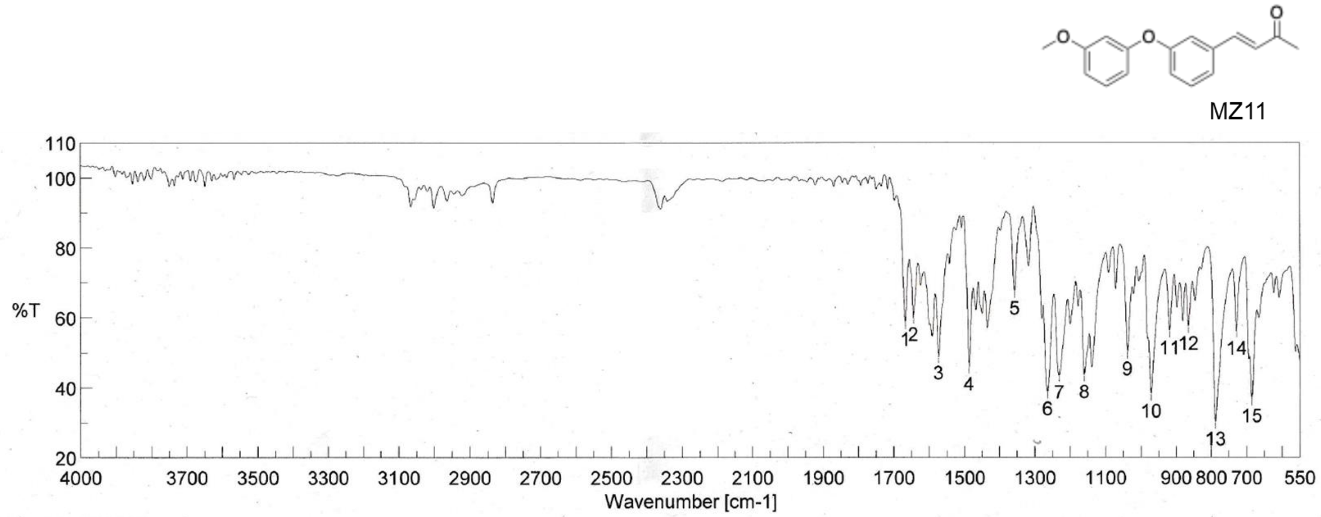

IR spectrum of MZ11.

- MZ12

2 steps yield 7%

1H-NMR (500 MHz, CDCl3) δ: 7.43 (d, J = 16.0 Hz, 1H), 7.31 (t, J = 8.0 Hz, 1H), 7.15-7.23 (m, 2H), 7.07 (s, 1H), 6.92-7.05 (m, 4H) , 6.62 (d, J = 16.0 Hz, 1H) , 3.82 (s, 3H) , 2.35 (s, 3H).
13C-NMR (126 MHz, DMSO-d6) δ:198.38, 158.59, 151.51, 144.14, 143.02, 135.91, 130.00, 127.53, 125.46, 122.39, 121.54, 121.20, 119.03, 115.90, 112.87, 55.89, 27.46.
HRMS-Esi: m/z [M-Na]$^-$ Calcd for $C_{15}H_{14}Na_1O_5$:297.07389, found: 297.07252. IR(ATR): 1668, 1487 cm^{-1}. Melting point: 55-56 ^{o}C.

MZ12
1H: MZ12_TO816B1_1HNMR

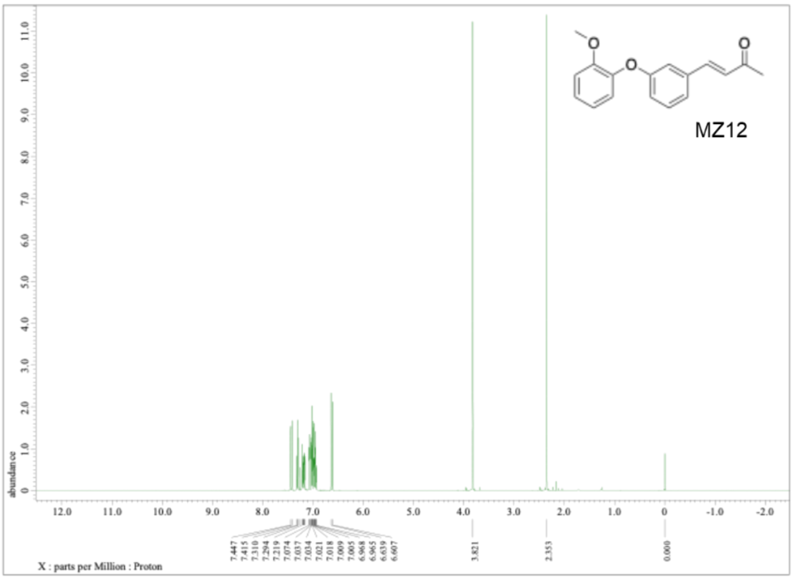

MZ12
13C: MZ12_TO816B1_13CNMR

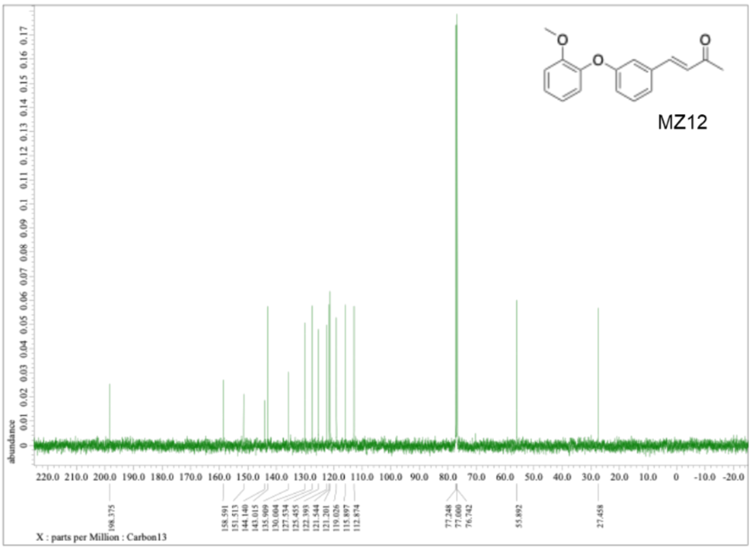

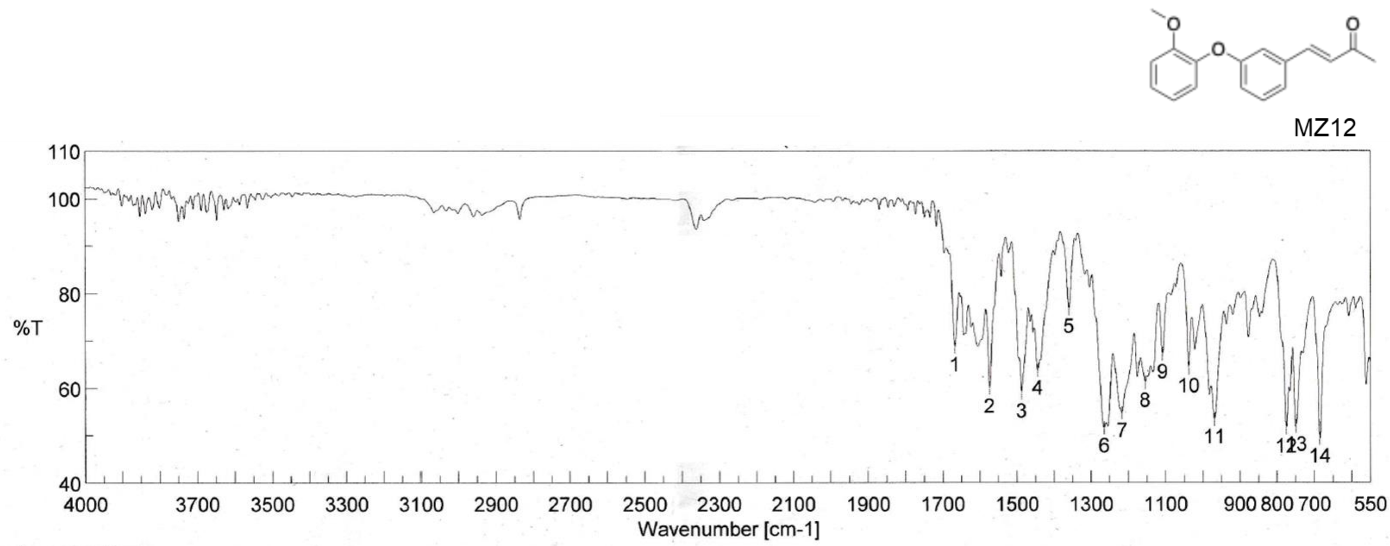

IR spectrum of MZ12.
